# Comparison of supine and prone miniaturized percutaneous nephrolithotomy in the treatment of lower pole, middle pole and renal pelvic stones: A matched pair analysis

**DOI:** 10.1590/S1677-5538.IBJU.2019.0049

**Published:** 2019-01-29

**Authors:** Akif Erbin, Harun Ozdemir, Murat Sahan, Metin Savun, Alkan Cubuk, Ozgur Yazici, Mehmet Fatih Akbulut, Omer Sarilar

**Affiliations:** 1 Department of Urology Haseki Traning and Research Hospital Istanbul Turkey Department of Urology, Haseki Traning and Research Hospital, Istanbul, Turkey

**Keywords:** Supine Position, Nephrolithotomy, Percutaneous, Pelvis

## Abstract

**Purpose:**

We aimed to compare the outcomes of supine and prone miniaturized percutaneous nephrolithotomy (m-PNL) in the treatment of lower pole, middle pole and renal pelvic stones.

**Materials and Methods:**

54 patients who performed supine m-PNL between January 2017 and March 2018 and 498 patients who performed prone m-PNL between April 2015 and January 2018 were included in the study. Of the 498 patients, 108 matching 1: 2 in terms of age, gender, body mass index, American Association of Anesthesiology score, stone size, stone localization and hydronephrosis according to the supine m-PNL group were selected as prone m-PNL group. The patients with solitary kidney, upper pole stone, urinary system anomaly or skeletal malformation and pediatric patients (<18 years old) were excluded from the study. The success was defined as ‘complete stone clearance’ and was determined according to the 1^st^ month computed tomography.

**Results:**

The operation time and fluoroscopy time in supine m-PNL was significantly shorter than prone m-PNL group (58.1±45.9 vs. 80.1±40.0 min and 3.0±1.7 min vs. 4.9±4.5 min, p=0.025 and p=0.01, respectively). When post-operative complications were compared according to the modified Clavien-Dindo classification, overall and subgroup complication rates were comparable between groups. There was no significant difference between the groups in terms of the success rates (supine m-PNL; 72.2%, prone m-PNL; 71.3%, p=0.902).

**Conclusions:**

Supine m-PNL procedure is more advantageous in terms of operation time and fluoroscopy time in the treatment of lower pole, middle pole and renal pelvic stones.

## INTRODUCTION

The main treatment modalities in urinary system stone disease are extracorporeal shockwave lithotripsy (ESWL), ureterorenoscopy (URS), percutaneous nephrolithotomy (PNL), open and laparoscopic surgery. With the recent advances in technology, endourologic procedures (URS and PNL) among the surgical treatments have gained more popularity. The European Association of Urology (EAU) urolithiasis guideline recommends standard PNL as the first choice in the treatment of kidney stones larger than 2cm (1). Although PNL is accepted as a safe method, it can lead to life-threatening hemorrhages. Considering that the hemorrhage in standard PNL is directly related to the instruments used, the diameters of the instruments have been reduced over the years. In this context, firstly, the miniaturized PNL (m-PNL) technique was introduced by Jackman et al. in 1988 (2). In the following years, developments have continued with defining smaller diameter systems such as ultra-m-PNL, super m-PNL and micro PNL techniques (3-5). The m-PNL is accepted as the use of 14-22Fr access sheaths by EAU Urolithiasis Guidelines Panel (6).

In PNL, the original position is accepted as ‘prone’. However, PNL can be performed in classic supine or different positions such as Galdacao modification of Valdivia, lateral decubitus, lateral position modification, and reverse lithotomy (7-10). Supine PNL was introduced by Valdivia in 1987 and the first results were reported in 1998 with a series of 557 cases (11, 12). When compared with the prone position, supine position has some advantages such as easier and comfortable patient positioning, possibility of simultaneous retrograde access to kidney, lower renal pelvic pressure and easier intervention to the respiratory tract by the anesthetist (13). There are many studies in literature comparing prone PNL and supine PNL, however, almost all of them are related to standard PNL. In the present study, our purpose was to compare the outcomes of supine and prone m-PNL performed for stones located in lower pole, middle pole and renal pelvis.

## MATERIALS AND METHODS

### Study design

The present study was approved by the Internal Institutional Review Board. Fifty four patients who performed supine m-PNL between January 2017 and March 2018 and 498 patients who performed prone m-PNL between April 2015 and January 2018 were included in the study.

Exclusion criterias were:

- pediatric patients (<8 years old)
- patients with solitary kidney
- patients with kidney stones located in the upper pole
- patients with urinary system anomalies
- patients with skeletal malformations

Of the 498 patients, 108 matching 1: 2 in terms of age, gender, body mass index (BMI), American Association of Anesthesiology (ASA) scores, stone size, stone localization and hydronephrosis (HN) according to the supine m-PNL group were selected as prone m-PNL group. Both groups were compared in terms of demographic data (age, gender, BMI, ASA score, previous surgery and ESWL), stone characteristics (size, localization, opacity, hydronephrosis), operative data (side, operation time, fluoroscopy time, number of access, size of access sheath, nephrostomy placement, transfusion, complication) and postoperative data (hospitalization time, hemoglobin drop, transfusion, JJ stent placement, success and complication). Operation time was calculated as the time from the insertion of ureteral catheter to nephrostomy placement. The success was defined as ‘complete stone clearance’ and was determined according to the 1^st^ month CT. Intraoperative complications were evaluated using the modified Stava classification system; postoperative complications were evaluated according to the modified Clavien-Dindo classification system (14, 15).

### Preoperative evaluation

Written and verbal consent was obtained from all patients before the operation. Patient assessment included medical history, physical examination, complete blood count, coagulation tests, serum biochemistry, urinalysis and urine culture. Anticoagulant drugs were discontinued at least 7-10 days week before the operation. All patients were evaluated preoperatively by non-contrast computed tomography (CT). Stone size was determined by measurement of the greatest dimension. In the case of multiple calculi, the sum of the greatest dimension of each stone was calculated. All patients had sterile urine culture prior to surgery. Antibiotic prophylaxis was provided by second generation cephalosporins. The first dose was administered intravenously when anesthesia was initiated and the second dose was given 12 hours later.

### Supine m-PNL technique

**Figure 1:**
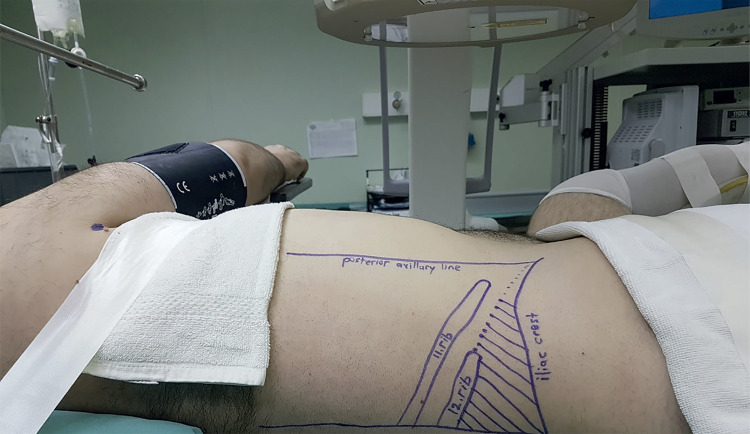
Galdakao-Modified Valdivia position in supine m-PNL. The shaded area between lower rib, posterior axillary line and iliac crest shows the subcostal access location.

Following general anesthesia, the patient was placed in the Galdakao-Modified Valdivia position. Under C-arm fluoroscopy guidance, 5 French (Fr) open end ureteral catheter was inserted retrogradely. A Foley catheter was then indwelled and the distal end of the ureteral catheter was fixed on the Foley catheter. Skin surface was marked to indicate the lower rib margin, posterior axillary line and iliac crest (Figure-1). The calyx plane to be punctured was determined by ultrasonography. Retrograde pyelography was done and an 18 gauge percutaneous access needle (Boston Scientific Corporation, Natick MA) was passed into the desired calix under fluoroscopic guidance. A 0.035 inch guidewire (Boston Scientific Corporation, Natick MA) was passed antegradely across the renal pelvis and into the ureter, upper or lower calix. The track was dilated sequentially using fascial and metallic dilators. According to stone sizes, the 15, 16.5 or 21Fr metallic sheats (Karl Storz, Tutlingen, Germany) were advanced over their metal dilators. A rigid 12Fr nephroscope (Karl Storz, Tuttlingen, Germany) was advanced through the sheath. Stone disintegration was achieved using a Holmium YAG Laser lithotripter (Sphinx, Lisa laser, USA). Flexible antegrade pyeloureteroscopy was performed if the rigid nephroscope couldn’t reach to stone. Stone fragments were removed with basket catheters. At the end of the procedure, retrograde pyelography was done to assess the integrity of the pelvicaliceal system (PCS). If there was no extravasation and irrigant fluid was returning clear, no tube was left (tubeless PNL); otherwise, a nephrostomy tube was left in place.

### Prone m-PNL technique

**Figure 2:**
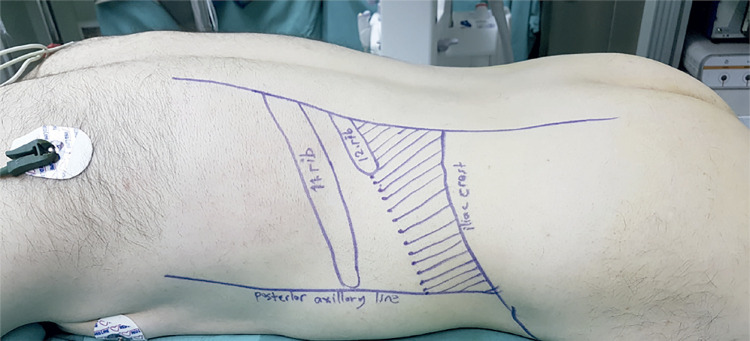
Prone position. The shaded area between lower rib margin, posterior axillary line and iliac crest shows the subcostal access location in prone PNL.

After the induction of general anesthesia, a 5Fr Ureteral catheter was placed and fixed on the Foley catheter in the lithotomy position. The patient was then repositioned in the prone position. Skin surface was marked to indicate the lower rib margin, posterior axillary line and iliac crest (Figure-2). Percutaneous access was achieved under C arm fluoroscopy guidance. The puncture was performed with an 18 gauge percutaneous access needle. Following successful puncture, a 0.035 inch guidewire was advanced through the needle into the PCS or ureter. At later stages, tract dilatation, nephroscopy, stone fragmentation, and stone retrieval were performed in a manner similar to supin m-PNL. All supine and prone procedures were performed by two experienced urologists at the tertiary referral center.

### Postoperative evaluation

A complete blood count and renal function tests were obtained from all patients within 6 hours after the operation. In cases with a nephrostomy tube, the tube was removed on postoperative day 1 or 2 after antegrade nephrostography revealed ureteral drainage down to the bladder. The leakage longer than 48 hours was accepted as ‘prolonged urine leakage’ and JJ stent was placed following CT imaging. JJ stents were removed under local anesthesia. All patients were evaluated with renal function tests and a non-contrast spiral CT 1 month after the operation.

### Statistical analysis

Data were analyzed by using Statistical Package for the Social Sciences software package version 20 (SPSS Inc., Chicago, IL, USA). Quantitative data were expressed as mean±std values on tables and categorical data were expressed with frequency (n) and percentages (%). The distribution of the variables was measured by the Kolmogorov Smirnov test. Independent t test was used to compare independent groups. Pearson Correlation test was used to examine the relationship between variables. Pearson Chi-Square and Fisher Exact tests were used to compare the categorical data. The data were analyzed at 95% confidence level and the threshold for statistical significance was accepted as p <0.05 for all analyses.

## RESULTS

Demographic data and stone characteristics are shown in Table-1. Age, sex, BMI, ASA score, stone size, stone localization and HN grade were similar between the groups because of fact that ‘1: 2 matched pair’ was performed.

**Table 1:** Demographic data and stone characteristics.

	Supine m-PNL (n=54)	Prone m-PNL (n=108)	p
Sex (female/male) *	15/39	38/70	0.343
Age (years)*	43.4±11.9	44.0±13.4	0.813
**BMI (kg/m**^2^)*	27.3±3.9	26.9±4.1	0.609
ASA score*	1.2±0.5	1.1±0.4	0.645
**Previous ESWL / surgery**			0.553
ESWL	9 (16.7%)	27 (25.0%)	
URS	2 (3.7%)	6 (5.6%)	
PNL	6 (11.1%)	16 (14.8%)	
Open Surgery	3 (5.6%)	6 (5.6%)	
**Stone opacity (opaque / non-opaque)**	50/4	94/14	0.289
Stone localization*			0.821
Lower calyx	8 (14.8%)	19 (17.6%)	
Middle calyx	0	0	
Upper calyx	2 (3.7%)	4 (3.7%)	
Pelvis	23 (42.6%)	51 (47.2%)	
Multiple calyx	21 (38.9%)	34 (31.5%)	
Stone size (mm)*	25.8±7.9	24.8±5.6	0.886
Hydronephrosis (mild/severe)*	43/11	85/23	0.891

* Matching parameters (1:2 scenario)

Operative data are summarized in Table-2. The operation time and fluoroscopy time in supine m-PNL was significantly shorter than prone m-PNL group (58.1±45.9 vs. 80.1±40.0 min and 3.0±1.7 min vs. 4.9±4.5 min, p=0.025 and p=0.01, respectively). While the rate of tubeless PNL was 37% in supine m-PNL group, it was 17.6% in prone m-PNL group (p=0.006). None of the patients had intercostal or upper pole access. There was no significant difference between the groups in terms of the intraoperative complications classified according to Satava.

**Table 2:** Operative data.

	Supine m-PNL (n=54)	Prone m-PNL (n=108)	p
**Operation side (right / left)**	27/27	51/57	0.739
**Operation time (min)**	58.1+45.9	80.1+40.0	0.025
**Fluoroscopy time (min)**	3.0±1.7	4.9±4.5	0.013
**Amplatz sheath size**			0.076
15 Fr	19 (35.2%)	21 (19.4%)	
16.5 Fr	20 (37.0%)	55 (50.9%)	
21 Fr	15 (27.8%)	32 (29.6%)	
**Access**			0.065
Lower pole	48 (88.9%)	85 (78.7%)	
Middle pole	6 (11.1%)	13 (12.0%)	
Multiple access	0	10 (9.3%)	
**Tubuless procedure**	20 (37.0%)	19 (17.6%)	0.006
**Intraoperative complication**			0.677
Satava grade 1a	2 (3.7%)	4 (3.7%)	
Satava grade 2a	1 (1.9%)	5 (4.6%)	

Post-operative complications and outcomes are summarized in Table-3. When post-operative complications were compared according to the modified Clavien-Dindo classification, overall and subgroup complication rates were comparable between groups. Grade-4 complications (angioembolization and urosepsis) were observed in 3 patients in both groups. The hospitalization time was similar and there was no significant difference between the groups in terms of the success rates (supine m-PNL; 72.2%, prone m-PNL; 71.3%, p=0.902). When success was separately evaluated as single stone and multicaliceal stone, there was no significant difference.

**Table 3:** Postoperative complications and outcomes.

	Supine m-PNL (n=54)	Prone m-PNL (n=108)	p
**Clavien - Dindo classification**			0.452
Grade 0	38 (70.4%)	76 (70.4%)	
Grade 1	5 (9.3%)	17 (15.7%)	
Grade 2	1 (1.9%)	5 (4.6%)	
Grade 3a	2 (3.7%)	3 (2.8%)	
Grade 3b	5 (9.3%)	4 (3.7%)	
Grade 4	3 (5.6%)	3 (2.8%)	
**Double-J stent placement**	1 (1.9%)	4 (3.7%)	0.521
**Fever**	3 (5.6%)	7 (6.5%)	0.817
**Hematocrit drop (gr/dL)**	3.9±3.3	3.2±3.0	0.376
**Transfusion**	4 (7.4%)	6 (5.6%)	0.644
**Angioembolization**	2 (3.7%)	1 (0.9%)	0.216
**Urosepsis**	1 (1.9%)	2 (1.8%)	0.214
**Hospitalization time (hour)**	56.3±62.5	66.0±37.2	0.401
**Overall success**	39 (72.2%)	77 (71.3%)	0.902
Success in isolated calyx stones	22 (66.7%)	53 (71.6%)	0.605
Success in multiple calyx stones	17 (81.0%)	24 (70.6%)	0.391

## DISCUSSION

In the literature, there is only one retrospective study comparing supine m-PNL and prone m-PNL (16). In our study, supine m-PNL and prone m-PNL were compared using ‘1: 2 match pair analysis’ in terms of success and complications. In the present study, general complication rates were similar in both group. Urosepsis was seen in one patient in the supine m-PNL group. Supin PNL provides the lower renal pelvic pressures. This is accepted as a protective factor for urosepsis (17). However, the presence of urosepsis in the supine m-PNL group suggested that the patient and operative factors (diabetes mellitus and long operation time) rather than surgical technique were effective in this patient.

In PNL, the pleura and the colon are the most injured organs. In the literature, supine and prone PNL have different numbers for colon injury. In the supine position, intestines will be more anteriorly displaced and this condition will reduce the risk of colonic injury (18). In a comparative study using the CROES database, colon injury was found to be similar in both groups (3.4% and 3.3%, p=0.95) (19). However, in the randomized prospective studies, no colonic injuries have been reported in the supine PNL (19-21). In our study, no organ injuries were detected in both groups. In the supine m-PNL group, no upper pole access was performed due to positional difficulty in patients. Antegrade or retrograde flexible ureterorenoscope were used in cases where upper pole access was required. Samely, in the unique study comparing supine m-PNL and prone m-PNL in the literature, no upper pole access was performed in the supine group (16).

In the meta-analysis including two randomized trials, it was reported that there was no statistically significant difference between supine standard PNL and prone standard PNL in terms of success rates (83.5% vs. 81.6%, respectively) (22). However, in another current meta-analysis, it was reported that prone standard PNL had significantly higher success rates than supine standard PNL (77.7% vs. 74.4%, p=0.0001). In the study, this difference was thought to be due to the fact that the nephroscope mobility was better in the prone PNL and that it was difficult to perform the upper pole access in the supine PNL (17). In the study comparing supine m-PNL and prone m-PNL, 54 and 126 patients were performed via supine m-PNL and prone m-PNL; the stone-free rates were 74.1% and 76.2%, respectively (16). Our study also confirmed that supine m-PNL and prone m-PNL were not superior to each other in terms of success.

We concluded that prone PNL procedure has a longer operation time than supine PNL. This difference is due to the time for repositioning the patient in prone PNL. In the meta-analysis study, it was reported that supine standard PNL had the advantage of an average operation time of 18 min and this difference was statistically significant (17). This result was also confirmed by a prospective randomized trial (21). In the study comparing supine m-PNL and prone m-PNL, operation times were 55 min and 82 min in supine m-PNL and prone m-PNL, respectively (16). In our study, the difference in operation time between supine and prone m-PNL was of average 22 minutes.

Because of the fact that it has some advantages in terms of cardiovascular, respiratory and anesthesia application, supine is a more accepted position by anesthetists than prone. There is a risk of the endotracheal tube being removed during the positioning to prone and the possibility of intervention to airway becomes limited after the patient is positioned. Furthermore, in the prone position, the risks of nerve tension, musculoskeletal injuries and visual impairment due to increased ocular pressure are more likely (23, 24). These risks are clinically insignificant in patients at low risk (ASA 1/2) groups (25). Despite its significant disadvantages, the prone position is used more often by surgeons. The reason for this is that surgeons are more accustomed to prone position. In the supine position, the surgeon can comfortably sit during the operation, and x-ray exposure is reduced because puncture and dilation of the nephrostomy tract are quite perpendicular to the body and the operator’s hands are outside the fluoroscopic field. Furthermore, by rotating the legs into the lithotomy position, combined antegrade and retrograde procedures can effectively be performed in the supine position. This represents the main advantage of this procedure because it combines the benefits of percutaneous and ureteroscopic intrarenal surgery in selected cases of contemporary treatment of bilateral stones (26).

Although the present study is a 1: 2 match pair analysis study, it has some limitations. The main limitations of the present study is its retrospective nature and the relatively small sample size. Thus, large-scale randomized trials should be encouraged to be designed, so that the above conclusions can be verified with an increased statistical power. Secondly, we did not match stone compositions for comparison. Theoretically, SFR could be affected by the differences in stone components between the two groups. Thirdly, we excluded the patients with skeletal malformations and with kidney stones located in the upper pole. As surface area used in prone m-PNL is extended, performing an access to the upper calyx is easier than supine m-PNL. In patients with wide hips and thin calices, it can be more difficult or even impossible to reach the upper calyx with a rigid nephroscope in supine position. So, the patients with upper pole stones were excluded from the study. Stone treatment in patients with skeletal deformity can be a serious problem for urologists. Skeletal deformities make both conventional and minimal invasive surgical interventions difficult. In these patients, it may be necessary to perform stone treatment by giving different positions other than supine or prone position. Also, for these patients, PNL may not always be the appropriate option. Instead, open surgery, laparoscopic-assisted PNL or f-URS may be more suitable options. Because of these reasons, the patients with skeletal deformity were excluded from our study. Another limitation of our study is that Guy’s stone score is not included. This system includes some parameters such as the presence of upper pole stone, anatomical abnormalities (calyceal diverticulum) and skeletal deformities (spina bifida, spinal injury). So, we were unable to use the Guy’s score in the present study.

## CONCLUSIONS

In the treatment of lower pole, middle pole and renal pelvic stones, supine m-PNL and prone m-PNL procedures have similar success rates. There is no significant difference in terms of general complication rates. However, supine PNL is more advantageous in terms of operation and fluoroscopy times.
